# Idiopathic stenosis of foramina of Monro in an asymptomatic adult patient: a rare entity radiologists should be aware of

**DOI:** 10.1259/bjrcr.20190102

**Published:** 2020-09-29

**Authors:** Natividad Gomez-Ruiz, Maria Carmen Polidura, Ana Maria Crespo Rodriguez, Juan Arrazola García

**Affiliations:** 1 Hospital Clínico San Carlos (Madrid), Madrid, Spain

## Abstract

Bilateral Adult Idiopathic Oclussion of Foramen of Monro is a rare entity, with less than 22 cases published in the literature so far, all of them symptomatic.^1^ When the symptoms require it, the current first-line treatment is endoscopic foraminoplasty, sometimes associated with septum pellucidum fenestration, although some authors consider that a more conservative treatment in paucisymptomatic patients.^2^ We report the case of an idiopathic biventricular hydrocephalus found incidentally in an asymptomatic 42-year-old female with temporomandibular joint disfunction. The fact that some patients with Monro foraminal stenosis may be asymptomatic increases the possibility of underdiagnosis, so we consider it a condition that radiologists should be aware of, mainly taking into account the fact that the diagnosis of this entity is usually radiologic^3^ and the potential complications associated with treatment.

## Clinical presentation

A 42-year-old female with clinical history of hypothyroidism and symptoms of right temporomandibular joint (TMJ) dysfunction was scheduled for a temporomandibular MRI scan, in which an enlargement of lateral ventricles was incidentally observed. She was then referred to the Neurology Department, where no signs of intracranial hypertension were found, nor history of headache, vomiting, dizziness, incontinence or gait disturbance, and a whole brain MRI study for hydrocephalus assessment was requested.

## Imaging findings

**Figure 1.**
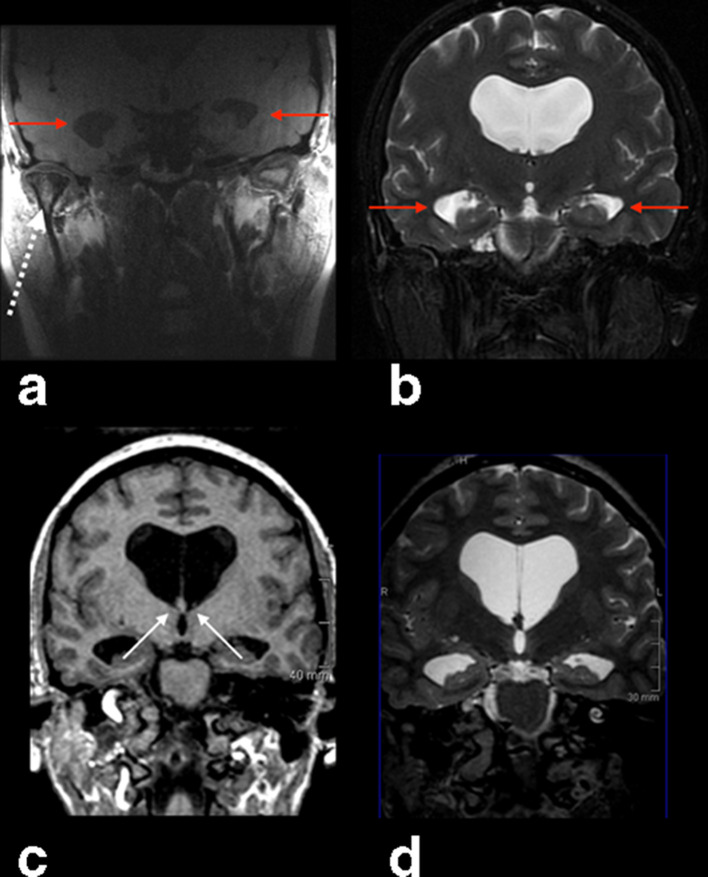
(a) Coronal FSE T1 of both TMJ obtained with specific surface coil shows signs of right TMJ degenerative arthropathy, such as flattening and decreased signal intensity of the mandibular condyle, osteophyte formation or thinning of the articular space (white dashed arrow). Enlargement of both temporal horns of the lateral ventricles were incidentally found (red arrows). (b) Coronal fat sat FSE T2 WI of the whole brain confirmed the enlargement of the lateral ventricles (red arrows). (c) 3D T1 FSPGR coronal plane reformat through Monro foramina showed bilateral stenosis (white arrows). (d) 3D CUBE T2 coronal reformat confirmed the stenosis. 3D, three-dimensional; FSE, fast spin echo; FSPGR, fast spoiled gradient echo; *T*2WI, *T2* weighted imaging; TMJ, temporomandibular joint.

**Figure 2.**
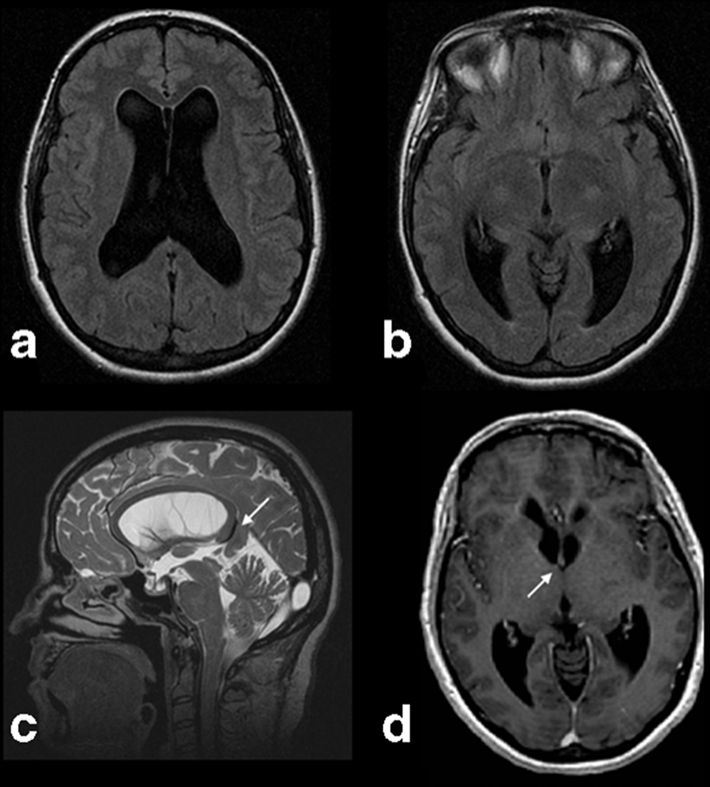
(a, b) Axial FSE *T*2 FLAIR WI of the whole brain shows normal periventricular signal intensity of the white matter and a slit-like third ventricle. (c) Sagital 3D CUBE *T*2WI confirmes the absence of dilatation or morphologic alterations of the third ventricle, along with a normal Silvio aqueduct. A subtle precuneus herniation due to focal tentorial hypoplasia was also noticed (white arrow). (d) Axial FSPGR 3D *T*1WI after i.v. contrast agent administration showed no abnormal enhancements. A stenotic foramina of Monro were found, mainly on the right side (white arrow). 3D, three-dimensional; FLAIR, fluid attenuated inversion recovery; FSE, fast spinecho; FSPGR, fast spoiled gradient echo; *T*1WI, *T1* weighted imaging; *T*2WI, *T2* weighted imaging.

Symmetric enlargement of the lateral ventricles and TMJ arthropathy were found on the MRI scan (Figure 1A). On the whole brain MRI study, the enlargement of the lateral ventricles was confirmed (Figure 1B), along with a silt-like third ventricle and a normal fourth ventricle (Figure 2B). No tumoral or cystic intraventricular lesions were present, and there were no signs of intraventricular hemorrhage or abnormal enhancement (Figure 2D). The periventricular white matter showed normal signal intensity on *T_2_* weighted sequences, and there were no significant brain herniations, except a subtle herniation of the precuneus, probable associated with focal tentorial hypoplasia (Figure 2C).

## Differential diagnosis

Enlargement of the lateral ventricles with slit-like third ventricle must raise the suspicion of idiopathic foramina of Monro stenosis. Nevertheless, secondary obstructive cause at or near the foramina should be firstly ruled out, since they are more frequent: hemorrhagic (*e.g.* SAH), inflammatory (ventriculitis), tumoral lesions (such as central neurocytoma) or tumor-like lesions and cysts (*e.g.* neurocysticercosis or colloid cyst) should be excluded, and the administration of i.v. contrast is usually necessary.

## Discussion

Foramen of Monro stenosis is a rare entity, with only a few cases published so far, all of them symptomatic. Four different types of stenosis have been described,^2^ depending on the unilateral or bilateral nature of the occlusion and the presence or absence of membranes. The most frequent symptoms found in these patients are headache and those associated with intracranial hypertension,^1^ none of them present in our case. We consider that patients with less severe or compensated stenosis and few or no symptoms are at risk of being underdiagnosed, mainly if radiologists don’t report adequately this disease when these patients have a brain CT or MRI done for other reasons. In order to avoid unnecessary invasive treatments, neurologists and neurosurgeons should also be aware of the existence of these asymptomatic cases, taking into account the possibility of complications associated to endoscopic approach, such as lesion of the fornix.

When a Monro foramina stenosis is suspected, MRI with i.v. contrast administration and three-dimensional high resolution sequences should be the technique of choice, as it allows a correct diagnosis and a better visualisation of potential membranes, and gives guidance for future surgical or endoscopical procedures.

## Learning points

1. Adult idiopathic bilateral stenosis of the Foramina of Monro is a rare cause of enlargement of the lateral ventricles.
2. Stenosis of the Foramina of Monro should be considered when a biventricular hydrocephalus with slit-like third ventricle is found, even in asymptomatic patients.
3. Radiological diagnosis of stenosis is done by exclusion after causes of obstruction, such as tumors or cysts, have been ruled out.
4. MRI with i.v. contrast agent administration and high resolution three-dimensional sequences should be the technique of choice for its diagnosis, to exclude inflammation or tumor, and to try to assess the presence of potential membranes as well as guide the endoscopic treatment, when indicated.
